# Deciphering the heterogeneity in DNA methylation patterns during stem cell differentiation and reprogramming

**DOI:** 10.1186/1471-2164-15-978

**Published:** 2014-11-18

**Authors:** Xiaojian Shao, Cuiyun Zhang, Ming-An Sun, Xuemei Lu, Hehuang Xie

**Affiliations:** Key Laboratory of Genomic and Precision Medicine, Beijing Institute of Genomics, Chinese Academy of Sciences, Beijing, 100101 China; Virginia Bioinformatics Institute, Virginia Tech, Blacksburg, VA 24060 USA; Department of Biological Sciences, Virginia Tech, Blacksburg, VA 24060 USA

**Keywords:** DNA methylation, Stem cell, Reprogramming, Cellular heterogeneity

## Abstract

**Background:**

Human induced pluripotent stem cells (iPSCs) have a wide range of applications throughout the fields of basic research, disease modeling and drug screening. Epigenetic instable iPSCs with aberrant DNA methylation may divide and differentiate into cancer cells. Unfortunately, little effort has been taken to compare the epigenetic variation in iPSCs with that in differentiated cells. Here, we developed an analytical procedure to decipher the DNA methylation heterogeneity of mixed cells and further exploited it to quantitatively assess the DNA methylation variation in the methylomes of adipose-derived stem cells (ADS), mature adipocytes differentiated from ADS cells (ADS-adipose) and iPSCs reprogrammed from ADS cells (ADS-iPSCs).

**Results:**

We observed that the degree of DNA methylation variation varies across distinct genomic regions with promoter and 5’UTR regions exhibiting low methylation variation and Satellite showing high methylation variation. Compared with differentiated cells, ADS-iPSCs possess globally decreased methylation variation, in particular in repetitive elements. Interestingly, DNA methylation variation decreases in promoter regions during differentiation but increases during reprogramming. Methylation variation in promoter regions is negatively correlated with gene expression. In addition, genes showing a bipolar methylation pattern, with both completely methylated and completely unmethylated reads, are related to the carbohydrate metabolic process, cellular development, cellular growth, proliferation, etc.

**Conclusions:**

This study delivers a way to detect cell-subset specific methylation genes in a mixed cell population and provides a better understanding of methylation dynamics during stem cell differentiation and reprogramming.

**Electronic supplementary material:**

The online version of this article (doi:10.1186/1471-2164-15-978) contains supplementary material, which is available to authorized users.

## Background

DNA methylation is the most common covalent modification known to occur on mammalian genomic DNA. During development, the establishment of tissue specific patterns of DNA methylation enables cells with same genetic composition to exhibit distinct phenotypes [1]. On the other hand, fully differentiated cells could be reprogrammed into pluripotent cells through different approaches, including nuclear transfer, cell fusion and transcriptional-factor transduction [2]. Epigenome remodeling is the key to these procedures to allow cells reacquiring pluripotency [3–5]. Besides dermal fibroblast, the inductions of iPSCs have been achieved with a number of human tissues. Adipose-derived stem cells (ADS) are a heterogeneous group of proliferative and multipotent mesenchymal stem cells. This cell population demonstrates differentiation capacity toward a variety of lineages, including adipogenic, chondrogenic, myogenic, neurogenic, and osteogenic cell lineages [6–9]. Considering the multipotency and tissue accessibility, ADS cells become one of the most attractive parental cells for reprogramming. Recently, great efforts have been made to improve the efficiency of iPSCs induction with ADS cells in a feeder-independent manner [10–13]. Providing the appropriate culture environment, these adipose-derived iPSCs exhibit the characteristics and morphologies similar to embryonic stem cells (ESCs).

Despite similar global gene expression and DNA methylation profiles to those of ESCs, iPSCs have been reported to frequently carry substantial genetic and epigenetic abnormalities [5, 14]. In normal cells, DNA methyltransferase 1 (DNMT1) is recruited to replication foci during DNA replication and faithfully copy the patterns of DNA methylation from the parental to the daughter DNA strand [15]. Remarkably accurate transmission of genomic DNA methylation patterns has been documented in both *in vitro* and *in vivo* studies [15–17]. However, the activities of DNMTs are dynamic and under the control of post-transcriptional regulations mediated by miRNAs [18] and a variety of post-translational modifications [19]. Incompetent epigenetic inheritance mechanism in pluripotent stem cells or iPSCs at the early passages frequently results in the aberrant DNA methylation [20, 21]. In addition, epigenetic reprogramming of iPSCs requires many rounds of cell division to erase epigenetic memory or to establish epigenetic states [22, 23]. During the gradual reprogramming of iPSCs with long-term passaging, stochastic *de novo* methylation followed by selection/fixation was shown to be critical for the formation of ESCs-like methylation profiles [20]. Not surprisingly, such DNA methylation dynamics could result in substantial variation in DNA methylation patterns within a population of stem cells or iPSCs [20, 24].

Many previous studies made the assumption that all cells within a tissue are with identical or greatly similar methylation patterns. However, in a mixed cell population, cells may demonstrate similar phenotypes but with distinct methylation patterns on genomic regions associated with cell specification. Moreover, the heterogeneity in cellular composition, leukocytes for instance, was recognized as an important confounding factor that could compromise the resulting interpretations for methylation studies [25, 26]. These findings emphasize the importance of examining the methylation pattern heterogeneity within a cell population or between different cell types. However, it remains unknown whether the methylation variation for a given genomic locus would change during differentiation and reprogramming. As an important regulator on gene expression, DNA methylation on promoters is negatively correlated with gene transcription [27]. Recently, the comparison on methylation levels of 69 human individuals showed a modest negative correlation between DNA methylation variation and gene expression variation [28]. Nevertheless, the relationship between the promoter methylation variation within a cell population and the expression levels of associated genes are poorly understood.

In this study, we developed a computational pipeline to systematically analyze the methylation variation within a cell population. We reanalyzed the single-base-resolution DNA methylation maps for ADS cells, mature adipocytes differentiated from ADS cells (ADS-adipose) and iPSCs reprogrammed from ADS cells (ADS-iPSCs) [5]. Specifically, we aim to gain global views on DNA methylation variation in cells with different levels of pluripotency, explore its relationships to different genomic features, and analyze the dynamics of promoter methylation pattern during differentiation and reprogramming.

## Results

**Figure 1:**
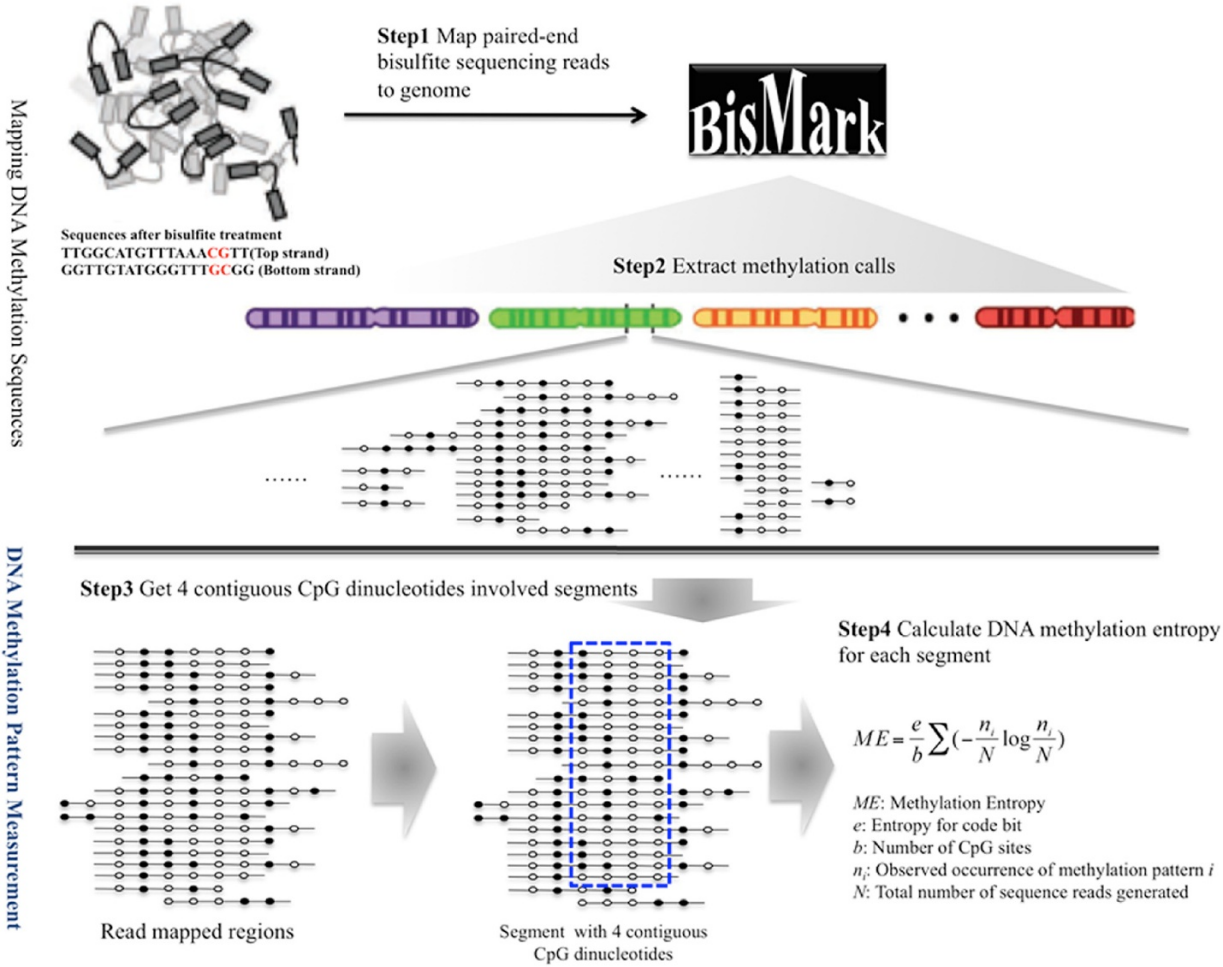
**Diagram of extracting DNA methylation pattern and calculation of entropy.** Step 1. Mapping paired-end DNA methylation reads to Human reference; Step 2. Extracting DNA methylation calls; Step 3. Obtaining 4 contiguous CpG dinucleotides involved segments; Step 4. Calculating DNA methylation entropy for each segment. Filled and open circles represent methylated and unmethylated CpG dinucleotides, respectively.

In order to determine DNA methylation variation within a cell population, we designed and implemented a data analysis pipeline illustrated in Figure 1. All bisulfite sequencing reads were first mapped to an artificially-bisulfite-converted reference genome with Bismark [29]. According to the mapping results, only reads with four or more CpG dinucleotides were kept and the methylation calls for CpG dinucleotides were extracted. We progressively scanned the entire methylome to identify genomic DNA segments with four neighboring CpG dinucleotides and at least sixteen read coverage. Using this analytical procedure we were able to reanalyze several recently published methylomes for ADS and derivatives [5, 30] to obtain a genome-wide view of DNA methylation variations during cell differentiation and reprogramming.

### Global assessment of methylation heterogeneity

A total of 1,097,779, 1,803,432 and 3,197,158 segments covering 2,506,221, 3,885,201 and 6,316,365 CpG sites were identified for ADS cells, ADS-adipose cells and ADS-iPSCs, respectively (Table 1). For each segment, the average DNA methylation level and the methylation entropy [31] were determined. To enable cross-sample comparison, further analyses were focused on the common set of 754,372 segments for three methylomes.

**Table 1:** **Statistics of MethylC-Seq data for the ADS, ADS-adipose and ADS-iPSCs**

Cell type	# Segments	# Total CpG sites (%)	# Sequence reads	Average reads per segment	Average level	Average entropy
ADS	1097779	2506221 (9%)	21678208	19.75	0.39	0.25
ADS-adipose	1803432	3885201 (14%)	36759110	20.38	0. 43	0.26
ADS-iPSCs	3197158	6316365 (23%)	73199400	22.90	0.60	0.21
Common segments	754372	1822209 (7%)	15412714	20.43	0.38	0.24
			16677071	22.11	0.38	0.24
			21202094	28.11	0.46	0.19

**Figure 2:**
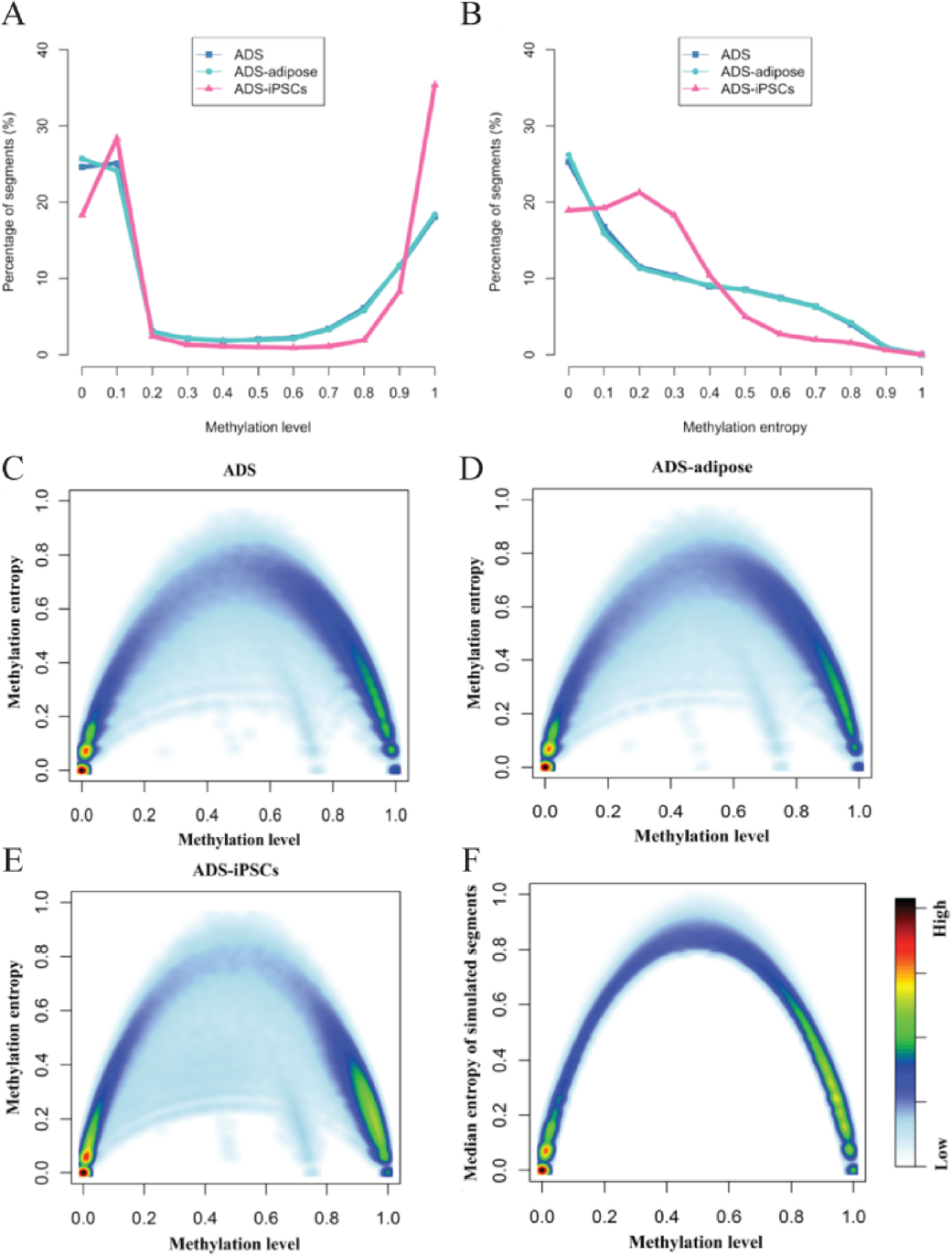
**Distribution of DNA methylation level and entropy for three methylomes.** The distribution was calculated based on the 754,372 common segments of all three methylomes. **(A)** DNA methylation level. **(B)** DNA methylation entropy. The DNA methylation level was plotted against the DNA methylation entropy for all three cell-types: ADS **(C)**, ADS-adipose **(D)**, and ADS-iPSCs **(E)**. The distribution of the median of the methylation entropies for simulated segments with 4 CpG sites was plotted **(F)**.

Similar to the previous observation [5], the methylation levels of CpG dinucleotides and the identified four-CpG segments follow a bimodal distribution (Figure 2A). For ADS, ADS-adipose and ADS-iPSCs, a total of 82.3%, 82.7% and 92.7% segments are either hypermethylated (average methylation level >80%) or hypomethylated (average methylation level <20%). The methylation profiles of ADS cells and ADS-adipose cells resemble each other, but are significantly different from that of ADS-iPSCs. Compared with ADS and ADS-adipose, ADS-iPSCs are with less hypomethylated segments (49.0% *vs.* 52.6% and 52.6%), especially for the completely unmethylated segments (18.3% *vs.* 24.6% and 25.7%) but with ~13% more hypermethylated segments. DNA methylation entropy is highly correlated to methylation level. According to the definition, the entropy of completely methylated or unmethylated segments is zero [31]. Approximately 26% of segments demonstrate homogenous methylation patterns with methylation entropy as zero in ADS and ADS-adipose cells and over 96% of these segments are completely unmethylated (Figure 2B). Maximum methylation entropy may be observed in half-methylated regions with highly variable methylation patterns. We plotted the methylation levels and entropy for the common set of four-CpG segments observed in three methylomes together with simulation result. The simulation was performed based on the read depth and methylation level of a given four-CpG segment [31]. For each segment, we randomly assigned the methylation state of the CpG site in each read but achieved the same methylation level as real data. For each of the given four-CpG segments observed in the three methylomes, we repeated this simulation 1,000 times to obtain 1,000 random methylation patterns with the same methylation level and then determined the median of methylation entropies of the simulation results. For the majority of the four-CpG segments, the methylation entropies observed in three methylomes are lower than that of simulated counterpart (Figure 2C-F). This indicates that the majority of methylation patterns in genome regions are not originated from stochastic methylation events but rather under substantial constraint.

For ADS-iPSCs, 19.0% of segments are with homogenous methylation patterns and 88.2% are with methylation entropy less than 0.4. However, the proportion of segments with methylation entropy over 0.4 in ADS and ADS-adipose cells exceeds 27.2%. To show such observations are not limited to the common set, we further performed pairwise comparison between shared segments among three methylomes. Stronger correlation between ADS and ADS-adipose than between ADS and ADS-iPSCs was observed with respective to the methylation level and entropy (Additional file 1: Figure S1). We noticed that the methylome data generated for ADS-iPSCs are of much higher sequencing depth compared with the other two methylomes (28Xs read coverage on average for ADS-iPSCs, 20Xs for ADS and 22Xs for ADS-adipose). To reduce the potential bias resulted from the difference in sequence read depth, for each segment, we randomly chose 71.4% reads (20/28) and simulated for 1,000 times. The average methylation entropy of simulation sets for ADS-iPSCs is 0.19, which is only 0.01 less than the average entropy obtained from real data. This indicated that the low methylation entropy for ADS-iPSCs cannot be simply explained with additional sequence reads. Due to the low reprogramming efficiency, ADS-iPSCs were derived from manually picked colonies rather than the entire pool of ADS cells [5]. We then questioned whether the variation in the methylation pattern of ADS-iPSCs is similar to that of embryonic stem cells (ESCs). We made use of the methylome of human H1 ESCs from previous study [30] (Additional file 2: Table S1). Interestingly, we found the methylation level of ADS-iPSCs is highly correlated to that of H1 and methylation entropy is modestly correlated (Pearson correlation r = 0.93 and r = 0.51, respectively, Additional file 1: Figure S1). It suggested that the ADS-iPSCs cells gain the ES-cell like DNA methylation patterns through reprogramming. In conclusion, compared with differentiated cells, ESCs and iPSCs are with more homogenous DNA methylation patterns on genome average.

### DNA methylation heterogeneity varies in different genomic regions

It has been shown that a great number of partially methylated domains in ADS cells become highly methylated in ADS-iPSCs and such methylation changes are not uniformly distributed across the entire genome [5]. In addition, a previous study focusing on representative loci demonstrated that the CpG islands (CGIs) are with higher methylation fidelity than those of repetitive elements, such as LINE repeats [32]. Using genome-scale methylome data, we examined the dynamics of methylation variations between different cell lines, and among different genomic regions including CpG islands, gene structures, and various types of repeats.

**Figure 3:**
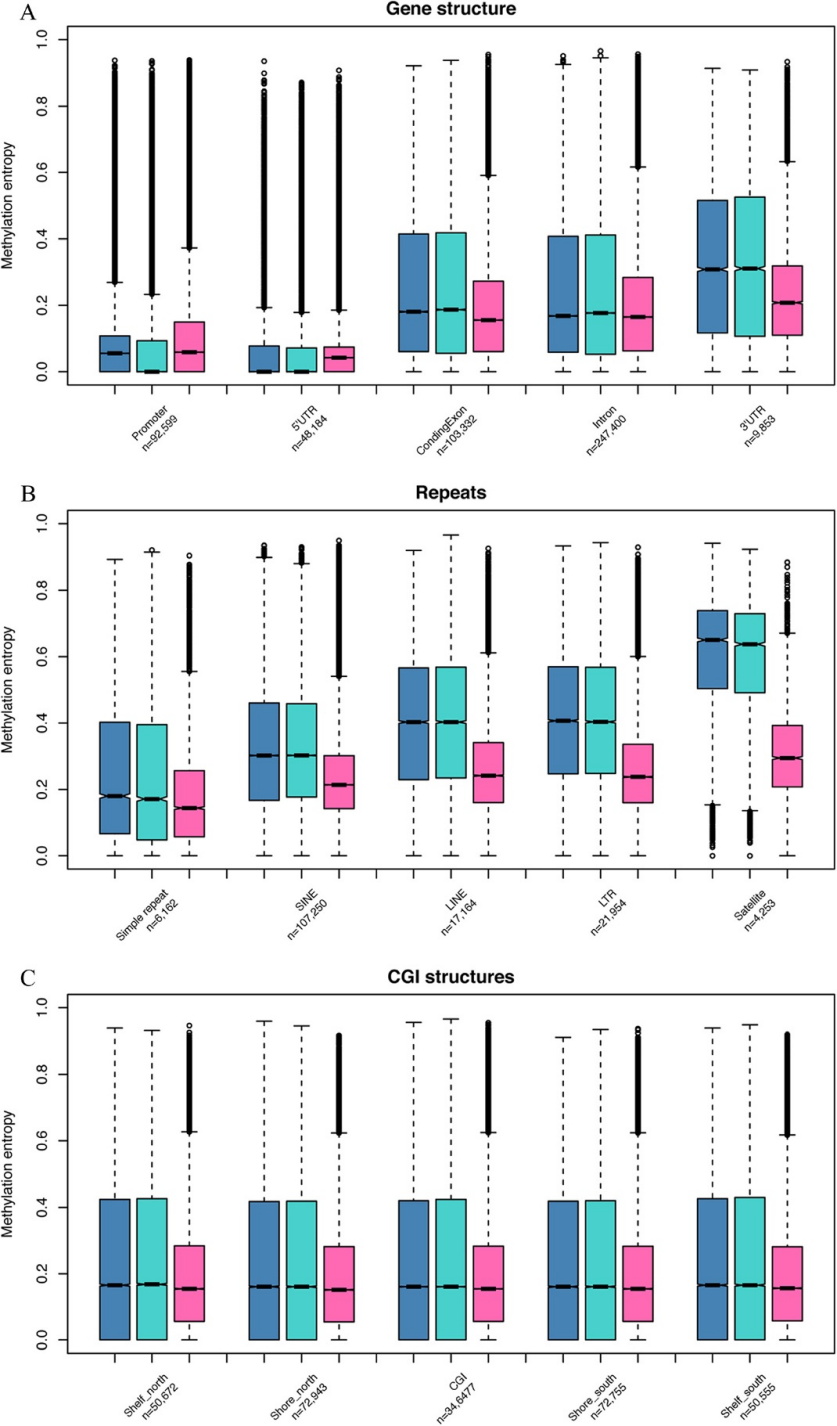
**DNA methylation entropy of different genomic regions for three methylomes.** The ADS, ADS-adipose and ADS-iPSCs (from left to right) were plotted in each box plot where shows the median, upper and lower quartiles and 95% confidence intervals. The number of segments within each class was shown below the class label. **(A)** methylation pattern variation in gene structures. **(B)** methylation pattern variation in repeat regions. **(C)** methylation pattern variation in CpG island structures.

According to the common set of three methylomes, the methylation entropies varied among different genomic regions (Figure 3). Compared with other gene-associated regions, promoters and 5’-UTRs show substantially low methylation variation (Figure 3A). Since CGIs are usually hypomethylated, we further classified promoters into two groups: CGI-promoters and non-CGI promoters. Interestingly, segments in CGI promoters show significantly homogeneous methylation patterns with the median methylation entropy near zero, whereas segments in non-CGI promoter show much higher methylation entropy (0.15, 0.14 and 0.20 for ADS, ADS-adipose and ADS-iPSCs, respectively). In addition, the median methylation entropies of coding exons, introns and 3’-UTRs decreased from 0.22 in ADS and ADS-adipose to around 0.17 in ADS-iPSCs. High methylation variation is observed in some repetitive elements. Satellite repeat regions show the highest methylation variation in all three methylomes while Simple repeats (micro-satellites) possess the lowest on average (Figure 3B). The degrees of DNA methylation variation among CGI shelves, CGI shores and CGI were similar in these three cell lines (around 0.16, Figure 3C). Intriguingly, ADS-iPSCs show higher methylation variation in promoters and 5’-UTR regions than those of ADS and ADS-adipose cells, but the lowest methylation variation in all the rest genomic regions.

### Methylation heterogeneity at TSS and bipolar methylated genes

**Figure 4:**
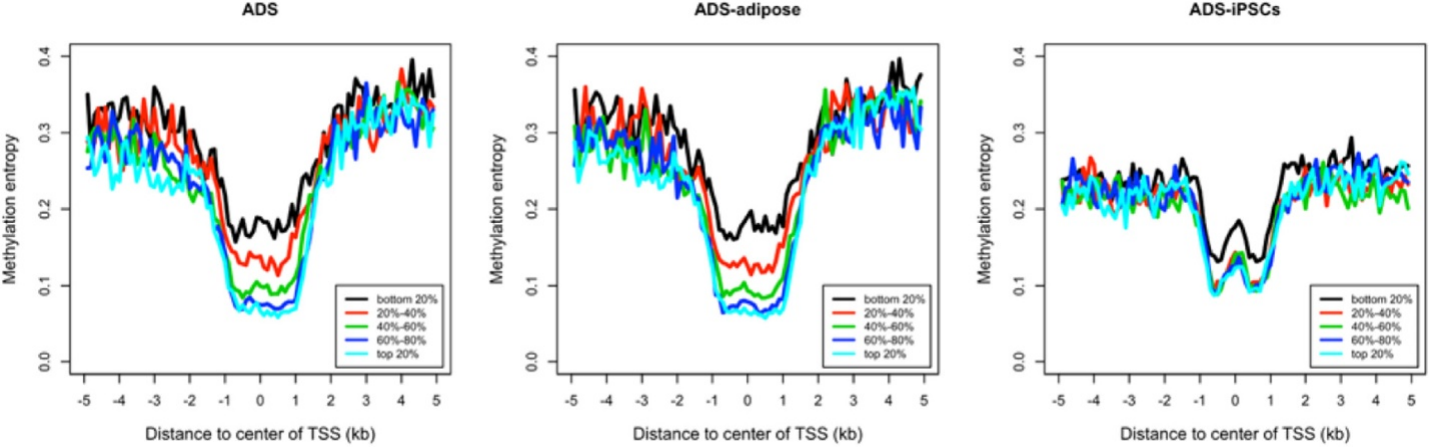
**Correlation of methylation entropy around the TSS regions with gene expression levels in ADS, ADS-adipose and ADS-iPSCs.** Genes were grouped as five equally-sized categories ranked by their expression levels.

DNA methylation on transcription start site (TSS) was known to be negatively correlated with gene expression in human stem cells [27]. Since methylation level is correlated with methylation entropy, we expect the degree of methylation variation may be associated with the level of gene expression as well. We re-analyzed the RNA-Seq data, and classified genes into five classes ranked by their expression levels. We found both the methylation level and entropy of segments around TSS are negatively correlated with gene expression in all three cell lines (Figure 4 and Additional file 3: Figure S2). For instance, in the ADS, the average methylation entropy is only 0.07 in promoters of the top expressed genes but reaches 0.19 in those of the lowest expressed genes. In general, the average methylation entropy decreases approaching TSSs. Interestingly, in ADS-iPSCs, we observed a small increase in the promoter regions immediately adjacent to the TSSs (Figure 4).

To further explore the epigenetic heterogeneity at promoter regions, we retrieved the putative cell-subset specific methylated segments as described in the Methods section. We first identified 113,860, 112,343 and 141,200 four-CpG segments with completely methylated and completely unmethylated reads in ADS, ADS-adipose and ADS-iPSCs, respectively. Segments associated with allelic DNA methylation and stochastic methylation events were filtered. Accordingly, 82,555, 77,617 and 117,305 segments were identified as putative cell-subset specific methylated. The overlapped segments were merged into regions and a total of 2,497, 2,447 and 3,866 segments or regions were mapped to the gene promoters. After filtering genes with less than 10 CpGs within the gene promoter, a total of 175, 143 and 396 genes in ADS, ADS-adipose and ADS-iPSCs methylomes were identified, respectively (Additional file 4: Figure S3 for the overlapping relationship of the three gene sets). Using Ingenuity Pathways Analysis software, we found that the cell-subset specific methylated genes shared in all the three cell lines are enriched in biological processes involved in cellular development, cell death and survival. Cell-subset specific methylated genes in ADS, ADS-adipose are enriched in pathways associated with cell cycle, cellular movement, cellular assembly, and cellular organization. Meanwhile, cell-subset specific methylated genes in ADS-iPSCs are enriched for cellular growth, cellular proliferation, carbohydrate metabolism, cell-to-cell signaling and interaction (Additional files 5: Table S2, Additional file 6: Table S3, Additional file 7: S4).

## Discussion

In this study, we implemented a new workflow to decipher DNA methylation variation on a genome-wide scale and performed a comprehensive analysis on the methylation heterogeneity for ADS cells, mature adipocytes differentiated from ADS cells and iPSCs reprogrammed from ADS cells. During cell differentiation and reprogramming, although the majority of segments didn’t show significant changes, several genes were observed to show dynamic changes (Additional file 8: Figure S4A-C). For instance, *ABHD8* showed decreased methylation level and methylation entropy during differentiation whereas the hox gene *HOXA5* showed decreased level and entropy during reprogramming. Compared with ADS and ADS-adipose, ADS-iPSCs show reduced methylation variation on repeat regions but increased methylation variation in promoter regions. Such increased variation in promoters of ADS-iPSCs may have resulted from either the random selection among the starting cell population or aberrant DNA methylation [33]. DNA methylation is reported to be anti-correlated with gene expression level. During cellular differentiation, DNA methylation can stably silence gene transcription [1]. Here, we showed that the DNA methylation pattern variations were also anti-correlated with gene expression levels. Interestingly, for the reprogrammed ADS-iPSCs, higher plasticity was observed around TSSs of transcripts, especially for the lowly expressed genes (e.g. *LSP1*, Additional file 8: Figure S4C). In line with that, the segments with an increased methylation level are frequently accompanied with an increase in methylation entropy. The stochastic methylation events may also account for the high plasticity of lowly expressed genes, which are frequently heavily methylated. Recent single cell analyses revealed much more heterogeneous gene expression in iPSCs than in ES cells [34]. The high variability of DNA methylation at promoter regions observed for iPSCs in this study may provide a mechanistic explanation.

Within a mixed cell population, the degree of DNA variation varies for genomic segments at different methylation levels. Highly variable methylation patterns were frequently observed at intermediate methylated loci. Compared with the simulated results representing random methylation events, some intermediate methylated loci were observed to be with significantly low entropy. The methylation profiles of these regions may be a result of cell-subset specific methylation or allele-specific methylation [35]. Furthermore, recent work on human adipose-derived stem cells lineage commitment demonstrated that several lineage-specific genes contained plastic methylation patterns [36], which indicated the intermediate methylation with low entropy at gene promoters may be potentially related to lineage commitment events. Our result indicates that besides allele-specific methylation, there exist putative cell-subset specific methylated regions. Moreover, a number of regions (612, 654 and 2,272 segments for ADS, ADS-adipose and ADS-iPSCs, respectively) demonstrate only two extreme methylation patterns: either completely methylated or completely unmethylated. We identified promoters hosting such bipolar methylated regions and found that there are more than twice the cell-subset specific methylated genes in ADS-iPSCs than in ADS and ADS-adipose. Gene function analysis revealed these genes are highly associated with the process of metabolic, cellular growth and proliferation. This result suggests the existence of sub-populations of ADS-iPSCs, which may be with diverse growth patterns. Future studies are highly desired to further explore the functional relevance of these cell-subset specific methylation regions.

In this study, we arbitrarily chose four-CpG segments with > = 16 reads as our analysis objectives. Less CpG sites will lead to much more segments but reduce the complexity of the variations while more CpG sites will reflect much more complexity of pattern variations but will leads to less segments since the minimum requirement of read coverage to cover all the possible combinations of DNA methylation states will be high (e.g. for a five-CpG segment, we may have to require 2^5 = 32 reads). This is a limitation of our current method on methylation variation analysis. With high read coverage data, more accurate estimation may be achieved to determine DNA methylation pattern variations.

## Conclusions

In this study, we used entropy to quantitatively assess the heterogeneity of DNA methylation in the methylomes of adult stem cells (ADS), fully differentiated cells (ADS-adipose) and reprogrammed cells (ADS-iPSCs). The methylation variation varies among different gene-related regions and different types of repetitive elements. Globally, DNA methylation heterogeneity decreases during reprogramming and ADS-iPSCs shows a higher methylation variation in promoters and 5’UTRs. In addition, in ADS-iPSCs, promoters are more frequently associated with putative cell-subset specific methylated regions. Taken together, our study provided new insights into the methylation dynamics during cell differentiation and reprogramming.

## Methods

### Re-analysis of genome-wide bisulfite sequencing datasets

All human datasets re-analyzed in this study were generated in previous studies [5, 30] from cell lines and deposited in public domain. This research involves no human subject or identifiable personal information, and thus no ethics approval is required. The high-throughput genome-wide bisulfite sequencing datasets were downloaded from The Salk Institute (http://neomorph.salk.edu/human_methylome/) where the mapped reads were reported. The methylome of human H1 embryo stem cell was generated with Illumina single-end (75 bp in length) sequencing strategy and the methylomes of female adipose-derived stem cells (ADS), adipocytes derived from the ADS cells (ADS-adipose) and ADS induced pluripotent stem cells (ADS-iPSCs) were generated with a paired-end (75 bp x 2) bisulfite sequencing approach [5, 30].

In order to extract the methylation pattern embedded in each sequence read, we re-mapped all the reads to the human reference genome (NCBI37/Hg19) with Bismark V1.0.5 [29]. To ensure the accuracy of the mapping result and methylation pattern extracted, no mismatch between the query sequence and the reference sequence was allowed, and only unique mapped reads were adopted. For all MethylC-Seq datasets, each sequence read was progressively scanned to identify genomic segments with four neighboring cytosines. For methylation entropy analysis, the four-CpG segments with at least sixteen sequence reads were selected for further analysis. The methylation entropy of each four-CpG segment was calculated as described previously [31, 37] and the methylation level of a four-CpG segment was defined as the percentage of methylated CpG sites.

### Genome association analysis

NCBI build 37/Hg19 genome annotations, including transcripts, repetitive elements and CpG islands (CGI), were downloaded from the UCSC Genome Browser [38]. Promoters were arbitrarily defined as regions 1 kb upstream of each RefSeq transcript. 5’UTR, coding exon, intron and 3’UTR were defined according to the RefSeq annotation table. Several major repetitive elements, including LINE, SINE, LTR, Satellite, and SimpleRepeat from the RepeatMasker annotation table were used. Flanking regions surrounding CpG islands, including CGI shore (2 kb flanking regions of CGI) and CGI shelf (the neighboring regions of the shore and up to 4 kb away from the CGI), were considered. When analyzing methylation pattern of genes, segments located at 1 kb upstream and 200 bp downstream of each RefSeq transcript were determined and used to calculate the average methylation level and entropy.

### Analysis of RNA-Seq data

RNA-Seq data were adopted from a previous study [5] and originally annotated based on Hg18 RefSeq table. We updated the transcripts annotation information to the Hg19 RefSeq annotation table by using LiftOver tools (an utility from UCSC Genome Browser: http://hgdownload.cse.ucsc.edu/downloads.html). Genes were grouped as five equally-sized categories according to their expression levels. The average methylation level and entropy of segments surrounding the TSSs were calculated for each category using 100 bp windows.

### Identification of putative cell-subset specific genes

The putative cell-subset specific genes were determined according to the following pipeline: i) select four-CpG segments with both completely methylated and unmethylated reads; ii) calculate their weighted entropy and *p*-*values*; iii) filter allele-specific methylation associated (four-CpG segments with at least 1 bp overlap with reported allele-specific methylation regions in [35]) and four-CpG segments associated with stochastic methylation events (*p-value* >0.05); iv) merge the remaining segments within 1 bp distance into larger regions; iii) associate to genes, promoters of which overlapped with the putative cell-subset specific regions (a promoter region was defined as regions within 1 kb upstream and 200 bp downstream from TSS) ; iv) the segments or regions within the same promoter region were further merged; v) filter genes with the number of CpGs within the promoter that are less than 10 (the average number of CpG sites for associated genes).

The calculation of weighted DNA methylation entropy is similar to the original methylation entropy, except that each pattern was weighted with the number of methylation state transitions (from methylated CpG to unmethylated one, and vice versa) observed in a four-CpG segment. The number of transitions for a complete methylated or an unmethylated segment is zero, and the maximum number of transitions for a four-CpG segment is 3. For simulation purposes, given a DNA methylation pattern, we randomly generated the distribution of methylation patterns based on its read depth and methylation level, and then calculated the weighted methylation entropy. We repeated this process 1,000 times, and the *p*-*value* was determined as the frequency of simulations with weighted entropy lower than observed one. The *p-value* threshold was set as 0.05.

### Gene functional analyses

In order to investigate the function of relevance of different gene sets, we generated the functional analyses through the use of QIAGEN’s Ingenuity Pathway Analysis (IPA®, QIAGEN Redwood City, http://www.qiagen.com/ingenuity). The *p-value* was used to indicate the significantly enrichment, and the threshold was set as 0.05.

### Availability of supporting data

The datasets supporting the results of this article are included within the article or additional files. Original data re-analyzed in this manuscript are deposited in NCBI’s Short Read Archive database under accession number: SRA023829.2 and SRP003529.

## Electronic supplementary material

Additional file 1: Figure S1: Pairwise comparisons between overlapped segments among different methylomes. The methylation level correlations between ADS and ADS-adipose, between ADS and ADS-iPSCs, between ADS-adipose and ADS-iPSCs, and between ADS-iPSCs and H1 were plotted in (A). The methylation entropy correlations between corresponding methylomes were plotted in (B). (PDF 2 MB)

Additional file 2: Table S1: Statistics of MethylC-Seq data between ADS-iPSCs and H1. (DOC 41 KB)

Additional file 3: Figure S2: Correlation of methylation levels around the TSS regions with gene expression levels in ADS, ADS-adipose and ADS-iPSCs. Genes were grouped as five equally-sized categories ranked by their expression levels. (PDF 96 KB)

Additional file 4: Figure S3: Venn diagram of putative cell-subset specific methylated genes in ADS, ADS-adipose and ADS-iPSCs. (PDF 35 KB)

Additional file 5: Table S2: ADS cell-subset specific methylation associated gene function analysis. (DOC 40 KB)

Additional file 6: Table S3: ADS-adipose cell-subset specific methylation associated gene function analysis. (DOC 40 KB)

Additional file 7: Table S4: ADS-iPSCs cell-subset specific methylation associated gene function analysis. (DOC 40 KB)

Additional file 8: Figure S4: Illustration of DNA methylation dynamics during differentiation and reprogramming. Regional view of DNA methylation profile of (A) *ABHD8* showing increased level and entropy during differentiation, (B) *HOXA5* showing decreased level and entropy during reprogramming, (C) *LISP1* showing increased level and entropy during reprogramming. (PDF 3 MB)

## Abbreviations

ADS: Adipose derived stem cells
iPSCs: Induced pluripotent stem cells
ESCs: Embryonic stem cells
TSS: Transcription start site.
