# Supplementary material for: Deciphering the heterogeneity in DNA methylation patterns during stem cell differentiation and reprogramming
Source: BMC Genomics. 2014 Nov 18;15(1):978. doi: 10.1186/1471-2164-15-978 (PMC4242552; doi:10.1186/1471-2164-15-978)
Supplement: Supplementary file 2 — Additional file 2: Table S1: Statistics of MethylC-Seq data between ADS-iPSCs and H1. (DOC 41 KB) [file 12864_2014_6666_MOESM2_ESM.doc]

**Supplementary Table S1.** Statistics of MethylC-Seq data between ADS-iPSCs and H1.

| Cell type | #Segments | #Total CpG sites | #Sequence reads | Average Reads per Segment | Average Level | Average Entropy |
| --- | --- | --- | --- | --- | --- | --- |
| ADS-iPSCs | 3197158 | 6316365 (23%) | 73199400 | 22.90 | 0.60 | 0.21 |
| H1* | 144339 | 438267(1.7%) | 3099272 | 21.47 | 0.86 | 0.26 |
| Common segments | 125567 | 383240 (1.4%) | 3756037  2713159 | 29.91  21.61 | 0.86  0.86 | 0.24  0.26 |

*: as a control to ADS-iPSCs;
