# Supplementary figures and images for: Deciphering the heterogeneity in DNA methylation patterns during stem cell differentiation and reprogramming
Source: BMC Genomics. 2014 Nov 18;15(1):978. doi: 10.1186/1471-2164-15-978 (PMC4242552; doi:10.1186/1471-2164-15-978)

ADS

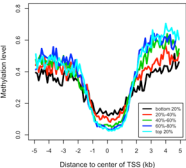

ADS-adipose

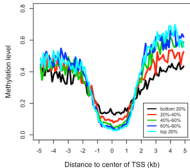

ADS-iPSCs

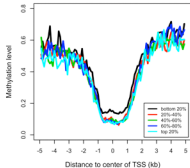

Supplement: Supplementary file 3 — Additional file 3: Figure S2: Correlation of methylation levels around the TSS regions with gene expression levels in ADS, ADS-adipose and ADS-iPSCs. Genes were grouped as five equally-sized categories ranked by their expression levels. (PDF 96 KB) [file 12864_2014_6666_MOESM3_ESM.pdf]

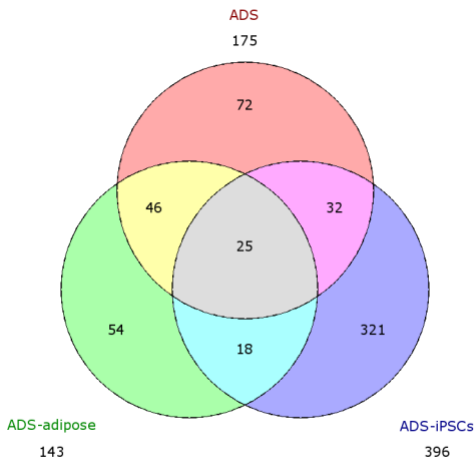

Supplement: Supplementary file 4 — Additional file 4: Figure S3: Venn diagram of putative cell-subset specific methylated genes in ADS, ADS-adipose and ADS-iPSCs. (PDF 35 KB) [file 12864_2014_6666_MOESM4_ESM.pdf]

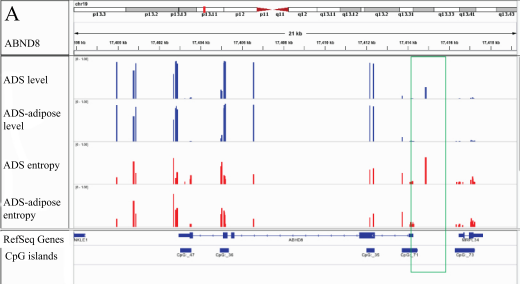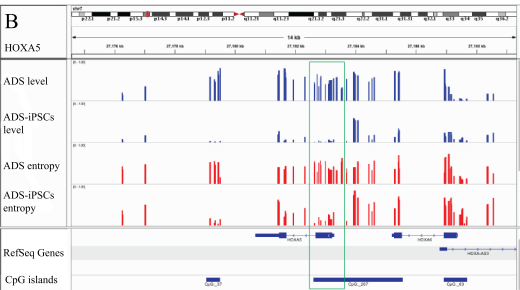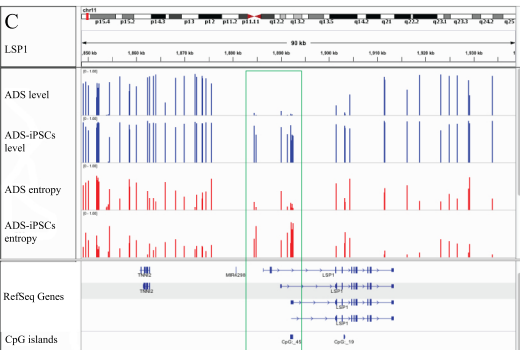

Supplement: Supplementary file 8 — Additional file 8: Figure S4: Illustration of DNA methylation dynamics during differentiation and reprogramming. Regional view of DNA methylation profile of (A) ABHD8 showing increased level and entropy during differentiation, (B) HOXA5 showing decreased level and entropy during reprogramming, (C) LISP1 showing increased level and entropy during reprogramming. (PDF 3 MB) [file 12864_2014_6666_MOESM8_ESM.pdf]
