# Supplementary material for: Deciphering the heterogeneity in DNA methylation patterns during stem cell differentiation and reprogramming
Source: BMC Genomics. 2014 Nov 18;15(1):978. doi: 10.1186/1471-2164-15-978 (PMC4242552; doi:10.1186/1471-2164-15-978)
Supplement: Supplementary file 5 — Additional file 5: Table S2: ADS cell-subset specific methylation associated gene function analysis. (DOC 40 KB) [file 12864_2014_6666_MOESM5_ESM.doc]

**Supplementary Table S2.** ADS cell-subset specific methylation associated gene function analysis.

| Category Term | Gene Count | P-Value |
| --- | --- | --- |
| Cell death and survival | 32 | 2.63E-04 - 3.51E-02 |
| Cellular development | 29 | 3.83E-04 - 3.80E-02 |
| Cellular growth and proliferation | 46 | 3.83E-04 - 3.80E-02 |
| Cell cycle | 19 | 8.54E-04 - 3.82E-02 |
| Cellular movement | 13 | 8.54E-04 - 3.19E-02 |
