# Supplementary material for: Deciphering the heterogeneity in DNA methylation patterns during stem cell differentiation and reprogramming
Source: BMC Genomics. 2014 Nov 18;15(1):978. doi: 10.1186/1471-2164-15-978 (PMC4242552; doi:10.1186/1471-2164-15-978)
Supplement: Supplementary file 6 — Additional file 6: Table S3: ADS-adipose cell-subset specific methylation associated gene function analysis. (DOC 40 KB) [file 12864_2014_6666_MOESM6_ESM.doc]

**Supplementary Table S3.** ADS-adipose cell-subset specific methylation associated gene function analysis.

| Category Term | Gene Count | P-Value |
| --- | --- | --- |
| Cellular assembly and organization | 14 | 8.55E-05 - 4.74E-02 |
| Cell death and survival | 39 | 1.05E-04 - 4.74E-02 |
| Cellular compromise | 8 | 1.70E-04 - 4.74E-02 |
| Cellular movement | 16 | 3.25E-04 - 4.74E-02 |
| Cellular development | 25 | 5.33E-04 - 4.95E-02 |
