# Supplementary material for: Deciphering the heterogeneity in DNA methylation patterns during stem cell differentiation and reprogramming
Source: BMC Genomics. 2014 Nov 18;15(1):978. doi: 10.1186/1471-2164-15-978 (PMC4242552; doi:10.1186/1471-2164-15-978)
Supplement: Supplementary file 7 — Additional file 7: Table S4: ADS-iPSCs cell-subset specific methylation associated gene function analysis. (DOC 40 KB) [file 12864_2014_6666_MOESM7_ESM.doc]

**Supplementary Table S4.** ADS-iPSCs cell-subset specific methylation associated gene function analysis.

| Category Term | Gene Count | P-Value |
| --- | --- | --- |
| Cellular development | 36 | 3.71E-04 - 3.17E-02 |
| Cellular growth and proliferation | 28 | 3.71E-04 - 3.17E-02 |
| Cell-To-Cell signaling and interaction | 20 | 4.45E-04 - 3.17E-02 |
| Carbohydrate metabolism | 15 | 7.55E-04 - 3.17E-02 |
| Cell death and survival | 18 | 7.55E-04 - 3.17E-02 |
