# Supplementary material for: Deciphering the heterogeneity in DNA methylation patterns during stem cell differentiation and reprogramming
Source: BMC Genomics. 2014 Nov 18;15(1):978. doi: 10.1186/1471-2164-15-978 (PMC4242552; doi:10.1186/1471-2164-15-978)
Supplement: Supplementary file 1 — Additional file 1: Figure S1: Pairwise comparisons between overlapped segments among different methylomes. The methylation level correlations between ADS and ADS-adipose, between ADS and ADS-iPSCs, between ADS-adipose and ADS-iPSCs, and between ADS-iPSCs and H1 were plotted in (A). The methylation entropy correlations between corresponding methylomes were plotted in (B). (PDF 2 MB) [file 12864_2014_6666_MOESM1_ESM.pdf]

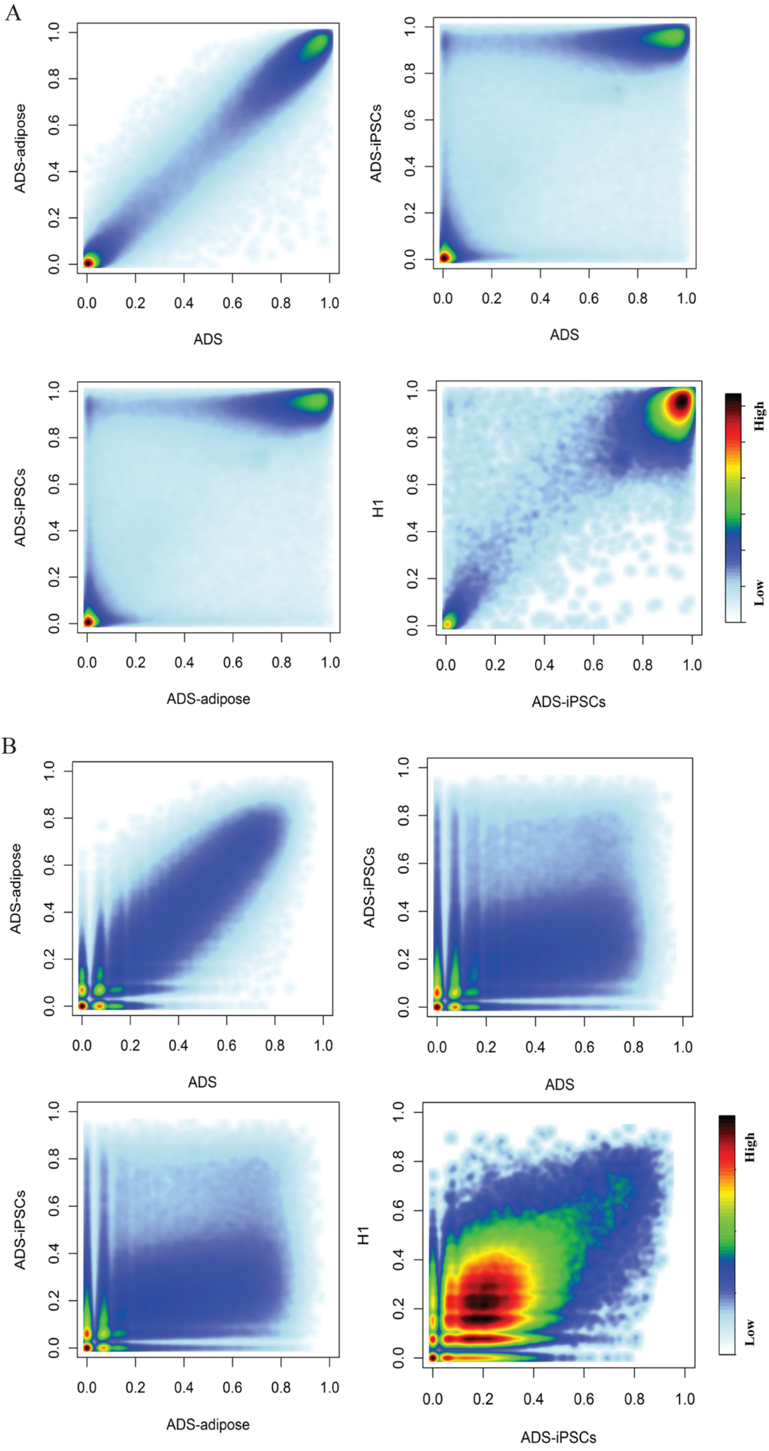
A
ADS-adipose
ADS
ADS-iPSCs
ADS
ADS-iPSCs
ADS-adipose
H1
ADS-iPSCs
High
Low
B
ADS-adipose
ADS
ADS-iPSCs
ADS
ADS-iPSCs
ADS-adipose
H1
ADS-iPSCs
High
Low
0.0
0.2
0.4
0.6
0.8
1.0
